# Patient Characteristics and Safety of Lecanemab in a Large Community‐Based Cohort at Sutter Health

**DOI:** 10.1002/alz70859_102433

**Published:** 2025-12-25

**Authors:** Armen Moughamian, Shawn Kile, Jane Kim, Aria Sengupta, Amanda Pickett, Julinna Cheung, Travis Urban, Chen Zhao, Lisamarie La Vallee, Govind Mukundan, Sylvia Sudat

**Affiliations:** ^1^ Ray Dolby Brain Health Center, Sutter Health, San Francisco, CA USA; ^2^ Sutter Neuroscience Institute – Memory Clinic, Sacramento, CA USA; ^3^ California Pacific Medical Center Research Institute, San Francisco, CA USA; ^4^ Sutter Health Neuroscience Service Line, Sacramento, CA USA; ^5^ Sutter Diagnostic Imaging, Sacramento, CA USA; ^6^ Sutter Health Center for Health Systems Research, Walnut Creet, CA USA

## Abstract

**Background:**

Lecanemab was the first disease modifying therapy for Alzheimer’s disease to receive full FDA approval but is associated with the serious side‐effect of ARIA (amyloid related imaging abnormalities). Due to the novelty of lecanemab and ARIA, uncertainty exists regarding the frequency and severity of ARIA in clinical practice. Here we summarize our experience with lecanemab in a community‐based healthcare system.

**Method:**

Data collection was approved by the Sutter Health IRB. All subjects were screened at a memory clinic. Eligibility for treatment was determined by the FDA labelling and Appropriate Use Recommendations were followed with a few exceptions. We present data on patients who received lecanemab between July 2023 and June 2024 since these patients received at least 6 months of lecanemab treatment, the highest risk time for ARIA.

**Result:**

210 patients were treated; the mean age was 75, 52% female, 70% mild cognitive impairment and 30% mild dementia. 37% were ApoE4 non‐carriers, 56% ApoE4 heterozygotes and 6% ApoE4 homozygotes. 36% of patients experienced infusions reactions (91% mild, 8% moderate and 1% severe). Lecanemab was discontinued in 14% of patients. There were 5 deaths, none attributed to lecanemab treatment. There were 33 cases (15.7%) of ARIA including ARIA‐E, ARIA‐H and ARIA‐E with ARIA‐H. 29 (13.8%) had ARIA‐H and 16 (7.6%) had ARIA‐E. ARIA occurred in 8 of 78 ApoE4 non‐carriers, 19 of 118 ApoE4 heterozygotes and 5 of 13 ApoE4 homozygotes. 8 cases of severe radiographic ARIA occurred (4 ApoE4 homozygotes, 3 ApoE4 heterozygotes and 1 ApoE4 non‐carrier). 3 cases of ARIA were symptomatic. A majority of ARIA occurred in the posterior cortex and ARIA‐H often occurred in the same region of ARIA‐E.

**Conclusion:**

Our data show that lecanemab can be safely delivered in a large community‐based healthcare system. Compared to the phase 3 CLARITY‐AD trial, we report higher rates of infusions related reactions but lower rates of ARIA. Our cohort contains fewer ApoE4 homozygotes (6%) compared to CLARITY‐AD (16%) which likely reduces our ARIA rates. However, we observe lower rates of ARIA in ApoE4 non‐carriers and ApoE4 heterozygotes compared to CLARITY‐AD, supporting that lecanemab can be safely administered in a community setting.

